# Real-Time Face Gesture-Based Robot Control Using GhostNet in a Unity Simulation Environment

**DOI:** 10.3390/s25196090

**Published:** 2025-10-02

**Authors:** Yaseen

**Affiliations:** Department of Electronics Engineering, Sejong University, Seoul 05006, Republic of Korea; yaseen@sju.ac.kr

**Keywords:** temporal facial gesture recognition, GhostNet-BiLSTM-Attention, human–robot interaction, touch-less control, Unity simulation, real-time recognition

## Abstract

Unlike traditional control systems that rely on physical input devices, facial gesture-based interaction offers a contactless and intuitive method for operating autonomous systems. Recent advances in computer vision and deep learning have enabled the use of facial expressions and movements for command recognition in human–robot interaction. In this work, we propose a lightweight, real-time facial gesture recognition method, GhostNet-BiLSTM-Attention (GBA), which integrates GhostNet and BiLSTM with an attention mechanism, is trained on the FaceGest dataset, and is integrated with a 3D robot simulation in Unity. The system is designed to recognize predefined facial gestures such as head tilts, eye blinks, and mouth movements with high accuracy and low inference latency. Recognized gestures are mapped to specific robot commands and transmitted to a Unity-based simulation environment via socket communication across machines. This framework enables smooth and immersive robot control without the need for conventional controllers or sensors. Real-time evaluation demonstrates the system’s robustness and responsiveness under varied user and lighting conditions, achieving a classification accuracy of 99.13% on the FaceGest dataset. The GBA holds strong potential for applications in assistive robotics, contactless teleoperation, and immersive human–robot interfaces.

## 1. Introduction

The use of robots, both mobile and stationary, in fields such as industrial automation, assistive technology, telepresence, entertainment, and scientific research has significantly increased in recent years [1]. With the rapid progress of Artificial Intelligence (AI) and computer vision, interactions between humans and robots have become increasingly common [2]. Among emerging methods, Intelligent Human–Robot Interaction (HRI) techniques are gaining attention as alternatives to conventional control systems [3]. Traditional approaches often rely on physical input devices such as joysticks or keyboards [4]. However, unlike these hardware-based interfaces, facial gesture-based HRI allows users to operate robots through intuitive and contactless expressions, making interaction more natural and accessible even for inexperienced users [5].

HRI research aims to create innovative designs and intuitive interfaces, typically grouped into four categories: wearable sensors, speech recognition, gesture-based systems, and user-friendly remote controls [6]. Table 1 summarizes the common advantages and disadvantages of typical HRI systems. In recent years, facial gesture-based recognition (FGR) systems have attracted growing interest due to the expressive and intuitive nature of facial movements as a means of communication [7,8]. Unlike hand gestures, facial gestures such as head tilts, eye blinks, or mouth movements can be performed with minimal effort, require no additional hardware, and remain effective even when the user’s hands are occupied, making them particularly suitable for contactless robot control [9].

Facial gesture recognition systems utilize various data sources, generally divided into two categories: sensor-based facial gesture recognition (S-FGR) [10] and vision-based facial gesture recognition (V-FGR) [11]. These categories differ primarily in their data acquisition techniques, data types, and training methodologies [12]. Sensor-based FGR typically employs specialized wearable devices or electrodes that capture muscle activity and subtle facial movements through electromyography (EMG) or inertial measurement units (IMUs) [13,14]. Such sensor data tends to be robust against external variations like lighting and background noise, and often requires less computational effort since the signals are directly obtained without complex image processing [15]. In contrast, vision-based FGR analyzes 2D images or video sequences captured by cameras, which makes it more accessible and cost-effective, as no additional hardware beyond a camera is needed [6,16]. Consequently, the majority of recent research has concentrated on V-FGR due to its simplicity and scalability [17].

Vision-based methods generally follow two approaches: extracting hand-crafted features [15] or employing deep learning to automatically learn features [10,18]. Hand-crafted methods typically utilize predefined facial landmarks, geometric features, or texture descriptors to recognize gestures [19]. Although these traditional approaches can be computationally efficient, they often lack adaptability to varying facial expressions and environmental conditions [20,21]. On the other hand, deep learning methods, particularly convolutional neural networks (CNNs), automatically learn hierarchical features from raw data, leading to superior accuracy and robustness [10,22]. However, these methods generally impose higher computational requirements and demand large annotated datasets for effective training [8,12,13].

Facial gestures can also be classified as either static or dynamic [12]. Static gestures involve holding a specific facial expression or pose for a certain duration [23], such as raising eyebrows or blinking, whereas dynamic gestures consist of a sequence of movements over time, like nodding or mouth movements [19]. While deep learning techniques have demonstrated excellent performance in recognizing static gestures due to their consistent visual patterns, dynamic gesture recognition remains more challenging [24]. The temporal nature of dynamic gestures adds complexity, resulting in an increased computational load and often lower accuracy compared to static gestures [25]. To address the temporal dimension of dynamic facial gestures, tracking algorithms combined with deep learning have been employed [9,26]. For instance, some works integrate pose estimation frameworks with tracking methods such as Kalman filters or DeepSORT [27] to extract and maintain consistent facial landmarks over video frames, facilitating temporal gesture classification. While these methods improve recognition robustness, they further increase computational complexity, which can hinder real-time performance [28].

Despite the advances in facial gesture recognition, many state-of-the-art methods [17,26,29,30] still struggle with balancing accuracy and efficiency. Real-time human–robot interaction systems require lightweight, fast, and reliable models that can operate under resource constraints without sacrificing safety or user experience [31]. To this end, several CNN and 3D CNN architectures [7,30,32] have been proposed, offering improved feature extraction and temporal modeling capabilities. However, these approaches typically entail high computational costs, motivating the search for efficient alternatives that maintain competitive performance [28,33].

To address these gaps, we propose GBA, a lightweight and efficient facial gesture recognition network that leverages the GhostNet feature extractor [34] and incorporates a BiLSTM with an attention mechanism [35]. This system accurately recognizes both static and dynamic facial gestures while maintaining low computational overhead. Furthermore, we developed a 3D robot simulation environment in Unity, enabling smooth and intuitive robot control through socket communication. This setup offers a faster and more user-friendly interface compared to previous approaches.

Overall, our novel approach leverages a streamlined set of facial gestures combined with a 3D simulation environment for remote robot operation. By integrating socket communication, the system functions as a virtual simulation platform, laying the groundwork for more advanced and contactless control methods. Compared to traditional joystick controllers and sensor-based interaction systems [3,12,21,31], our proposed solution offers improved speed, reliability, and user-friendliness. We summarize our main contributions in this work as follows:

- GhostNet-based spatial features achieve 99.13% accuracy (↑4.3%) at 30 FPS with only 1.1 GFLOPs, proving suitable for real-time use on embedded GPUs.
- Incorporated LSTM networks capture sequential dependencies in gesture patterns, improving robustness to temporal variations.
- Integrated spatial and temporal attention modules to focus on the most salient facial regions and critical time frames, boosting classification accuracy.
- Designed a Unity3D-based virtual environment where recognized facial gestures are converted into robot control commands via socket communication for interactive operation.
- Developed a fully trainable architecture that unifies spatial feature extraction, temporal modeling, and attention in a single efficient framework for human–robot interaction.

Furthermore, the related work on facial gesture recognition is reviewed in Section 2, followed by a detailed description of our proposed method in Section 3. The experimental setup, results, and analysis are presented in Section 4 with further discussion in Section 5. Finally, Section 6 concludes the paper and outlines future research directions.

**Table 1:** Human–robot interaction systems can be classified into several types, as outlined in [3,12,21,31].

HRI Method	Pros	Cons
Remote Control Devices	Familiar interface Low latency Precise control	Requires physical devices Limited mobility
Wearable Tracking Sensors	Accurate motion tracking Enables continuous monitoring	Intrusive Costly hardware User discomfort
Voice-Based Interaction	Hands-free interaction Intuitive for commands	Sensitive to noise Language dependent
Facial Gesture Interface	Contactless Intuitive and expressive No additional hardware needed	Sensitive to lighting Limited gesture vocabulary Computationally intensive

## 2. Related Work

Various methods have been proposed for facial gesture-based interaction in autonomous system control, employing different sensing modalities to improve recognition accuracy, responsiveness, and robustness in real time [36]. Sensor-assisted systems utilize data from IMUs, depth sensors, or motion capture devices to capture and localize subtle facial movements precisely [37,38]. Vision-based approaches, on the other hand, leverage RGB data from standard cameras, while some hybrid frameworks integrate both sensor and vision modalities to enhance recognition under challenging conditions [39,40]. In general, facial gesture recognition methods can be categorized into two main types: sensor-assisted facial gesture recognition (S-FGR) and vision-based facial gesture recognition (V-FGR).

### 2.1. Sensor-Assisted Facial Gesture Recognition

S-FGR techniques often employ wearable or external sensors to track head orientation, facial muscle activation, or eye movement patterns. In some approaches, inertial measurement units (IMUs) mounted on headgear or eyeglass frames record orientation changes to detect gestures such as nods or tilts [41]. Similarly, depth and 3D facial scanning devices can capture high-fidelity geometric changes in facial expressions, enabling more precise gesture classification [37]. Motion capture systems have also been employed to track facial landmarks in three dimensions, providing robustness against lighting changes and partial occlusions [42,43].

Machine learning algorithms such as support vector machines (SVMs) [44], decision trees (DTs), and k-nearest neighbors (KNNs) have been used extensively with sensor-based features to perform the classification of facial gestures. These methods are computationally efficient and can perform well in constrained environments; however, they typically require specialized hardware and are less suitable for unconstrained, everyday usage. More recent studies have explored hybrid approaches, combining electromyography (EMG) sensors with visual data to capture both muscle activity and external appearance changes [45], although these systems face challenges such as user discomfort and calibration requirements.

### 2.2. Vision-Based Facial Gesture Recognition

In vision-based FGR, facial gestures are detected directly from camera images or video streams, which can be considered a specialized case of facial expression recognition [40,46]. These approaches generally fall into handcrafted feature-based and deep feature-based categories. Handcrafted methods rely on manually designed descriptors such as local binary patterns (LBPs) or the histogram of oriented gradients (HOG) applied to facial regions [39], whereas deep learning-based automatically approaches extract discriminative features using convolutional or recurrent neural networks.

Deep feature-based methods have recently achieved state-of-the-art performance in FGR, with architectures including 2D CNNs [29,47], 3D CNNs for spatiotemporal modeling [12,48], recurrent models such as LSTMs for temporal dynamics [17,49], and hybrid transfer learning models leveraging pretrained networks such as VGG19, ResNet-50, and MobileNet [50,51,52]. Vision Transformers (ViTs) have also been explored for facial expression and gesture recognition [18,53], although their high computational cost currently limits their adoption in low-latency applications. For example, Aouayeb et al. [54] proposed a ViT enhanced with a squeeze-and-excitation (SE) block for facial expression recognition, achieving 99.8% accuracy on the CK+ dataset. While this demonstrates the strong discriminative capacity of transformer-based models, the evaluation on a relatively small and controlled dataset suggests potential challenges in generalizing to more diverse, in-the-wild scenarios. Building on this line of work, Wasi et al. [55] introduced ARBEx, a framework for facial expression learning that leverages Vision Transformers. The framework combines several strategies to mitigate common challenges in facial expression learning, including class imbalance, bias, and uncertainty, highlighting the potential of transformer-based approaches to enhance robustness in expression recognition tasks.

More recently, Li et al. [56] proposed FER-former, a Transformer-based architecture for facial expression recognition in the wild that employs multifarious supervision. The model integrates multi-granularity embeddings, a hybrid self-attention mechanism, and domain-steering supervision to improve feature learning. By combining features from both CNNs and Transformers through a hybrid stem, FER-former achieves enhanced representation for FER tasks. Experiments across multiple benchmarks demonstrate that this approach outperforms several state-of-the-art methods, highlighting the benefits of hybrid CNN-Transformer designs for in-the-wild expression recognition. Similarly, Xue et al. [57] proposed TransFER, which integrates Multi-Attention Dropping and Multi-head Self-Attention mechanisms to capture rich, relation-aware local features for FER. While effective, it was slightly outperformed by FER-former [56] across multiple benchmarks but still surpassed other state-of-the-art methods.

Finally, Huang et al. [58] proposed a CNN-based framework with a dual attention mechanisms: grid-wise attention for low-level feature extraction and a visual Transformer for high-level semantic representation. It achieved up to 99.0% accuracy on CK+ but has not been validated extensively on larger, diverse datasets or optimized for lightweight, real-time deployment.

In summary, while these Transformer- and attention-based methods achieve high accuracy, they often suffer from high computational cost, limited generalization to unconstrained environments, and challenges for real-time HCI deployment. Significant progress has been made in both sensor-assisted and vision-based FGR. Still, real-time deployment in interactive systems, especially in immersive environments, remains challenging due to factors such as varying lighting, diverse user characteristics, and the need for low-latency inference [3,6,26]. To advance the state of the art in practical applications, we propose GBA, a lightweight vision-based FGR system integrated into a 3D Unity simulation environment for real-time robot control. The system operates without specialized sensors, mapping recognized facial gestures to control commands transmitted over network sockets between machines, enabling a seamless and contactless human–robot interaction experience.

## 3. Methodology

**Figure 1:**
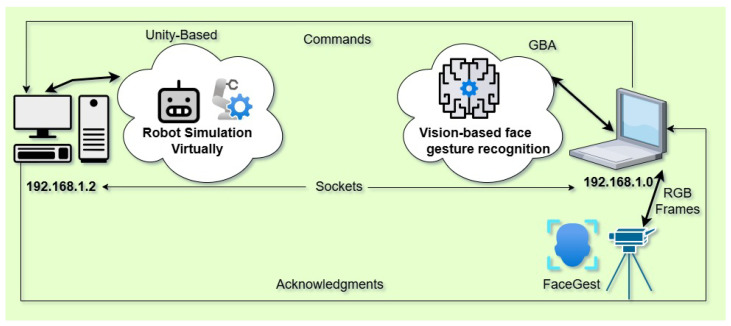
Gesture-based robot control: Real-time facial gesture recognition for interactive robot navigation in a unity simulation via sockets.

This section presents an integrated 3D virtual environment system for controlling a ground robot, which communicates in real-time with the GBA (FGR module) via TCP/IP sockets. The two modules, the GBA and the 3D Unity simulation, are connected through network protocols, enabling intuitive and interactive robot control using facial gestures, as illustrated in Figure 1.

### 3.1. Problem Formulation

Given a video sequence of face images $X=\{x_{1},x_{2},...,x_{T}\}$, where $x_{t}\in R^{H\times W\times C}$ is the face image at time step *t*, the goal is to classify the sequence into one of *K* discrete face gesture classes.

Formally, the task is to learn a mapping

(1) f:X→y,y∈{1,2,...,K}

that accurately predicts the gesture label *y* based on spatiotemporal patterns.

### 3.2. Spatial Feature Extraction with GhostNet

Each frame $x_{t}$ is passed through a GhostNet convolutional backbone to extract a compact spatial feature vector $f_{t}\in R^{d}$.

#### 3.2.1. GhostNet Backbone

**Figure 2:**
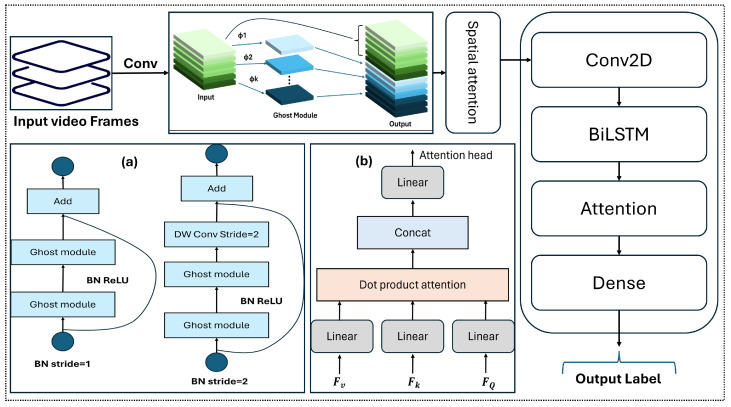
Architecture of the proposed GhostNet-BiLSTM with attention (GBA) model, illustrating spatial feature extraction with GhostNet, spatial and temporal attention mechanisms, and temporal modeling via BiLSTM. Panel (**a**) shows GhostNet bottlenecks with strides 1 and 2; and panel (**b**) depicts the attention heads.

GhostNet is designed to generate efficient feature maps by combining intrinsic features with cheap linear transformations, reducing computational cost. Formally, GhostNet computes the feature maps as

(2) $$F=\mathrm{GhostModule}\left(x_{t}\right)=\left[f^{\left(1\right)},f^{\left(2\right)},...,f^{\left(d\right)}\right]$$

where each $f^{\left(i\right)}$ is generated by a standard convolution and refined from intrinsic features using efficient linear operations. The GhostNet module performs spatial feature extraction, which is enhanced with spatial attention. These features are then processed by a BiLSTM for temporal modeling, followed by a temporal attention mechanism. Figure 2 illustrates this pipeline: (a) shows the GhostNet bottlenecks with strides 1 and 2; and (b) shows the attention heads.

#### 3.2.2. Spatial Attention Module

To enhance the representational capacity, a spatial attention mechanism is integrated after the GhostNet backbone to focus on the most informative facial regions.

The spatial attention map $A_{t}\in {\left[0,1\right]}^{H^{\prime }\times W^{\prime }}$ is computed as

(3) $$A_{t}=\sigma \left(f_{att}\left(F_{t}\right)\right)$$

where $f_{att}$ is a convolutional layer followed by a sigmoid activation σ(·), and $F_{t}$ is the spatial feature map tensor before pooling.

The attended feature $F_{t}^{att}$ is then

(4) $$F_{t}^{att}=A_{t}⊙F_{t}$$

where ⊙ denotes element-wise multiplication.

Finally, global average pooling is applied to get the spatial feature vector:

(5) $$f_{t}=\mathrm{GAP}\left(F_{t}^{att}\right)\in R^{d}$$

### 3.3. Temporal Modeling with Bidirectional LSTM and Attention

The extracted spatial features $f_{t}$ from each frame are concatenated into a sequence:

(6) $$F=\{f_{1},f_{2},...,f_{T}\}$$

which is input to a Bidirectional Long Short-Term Memory (BiLSTM) network to model temporal dependencies in face gestures.

#### 3.3.1. Bidirectional LSTM

A standard LSTM cell computes hidden state $h_{t}$ and cell state $c_{t}$ at time step *t* as

(7) $$\begin{array}{cc} i_{t} & =\sigma (W_{i}f_{t}+U_{i}h_{t-1}+b_{i})\\ f_{t}^{gate} & =\sigma (W_{f}f_{t}+U_{f}h_{t-1}+b_{f})\\ o_{t} & =\sigma (W_{o}f_{t}+U_{o}h_{t-1}+b_{o})\\ g_{t} & =\tanh(W_{g}f_{t}+U_{g}h_{t-1}+b_{g})\\ c_{t} & =f_{t}^{gate}⊙c_{t-1}+i_{t}⊙g_{t}\\ h_{t} & =o_{t}⊙\tanh\left(c_{t}\right) \end{array}$$

In a bidirectional setting, two LSTM layers process the sequence forwards and backwards, and their outputs are concatenated:

(8) $$h_{t}^{bi}=\left[\vec{h_{t}};\overset{\leftarrow }{h_{t}}\right]$$

#### 3.3.2. Temporal Attention Mechanism

To emphasize important time steps in the gesture sequence, a temporal attention mechanism is applied over the BiLSTM outputs $H=\{h_{1}^{bi},...,h_{T}^{bi}\}$.

The attention weights $\alpha_{t}$ are computed as

(9) $$e_{t}=v^{⊤}\tanh\left(W_{h}h_{t}^{bi}+b_{h}\right)$$

(10) $$\alpha_{t}=\frac{\exp(e_{t})}{\sum_{k=1}^{T}\exp\left(e_{k}\right)}$$

where v, $W_{h}$, $b_{h}$ are learnable parameters.

The context vector c representing the entire sequence is

(11) $$c=\sum_{t=1}^{T}\alpha_{t}h_{t}^{bi}$$

### 3.4. Classification Layer

The context vector c is passed through fully connected layers with nonlinearities to predict the gesture class probabilities:

(12) $$p=\mathrm{Softmax}(W_{c}c+b_{c})$$

where $p\in R^{K}$ represents the predicted class probabilities.

The predicted label is

(13) $$\hat{y}=\arg\max_{k}p_{k}$$

### 3.5. Robot Control via Unity Simulation with Socket Communication

To realize interactive robot control in a metaverse-like environment, the predicted gesture $\hat{y}$ is converted to a control command and sent in real-time to the Unity 3D simulation via socket communication. The messaging protocol, key parameters, and gesture–command associations are summarized in Table 2 and Table 3.

**Figure 3:**
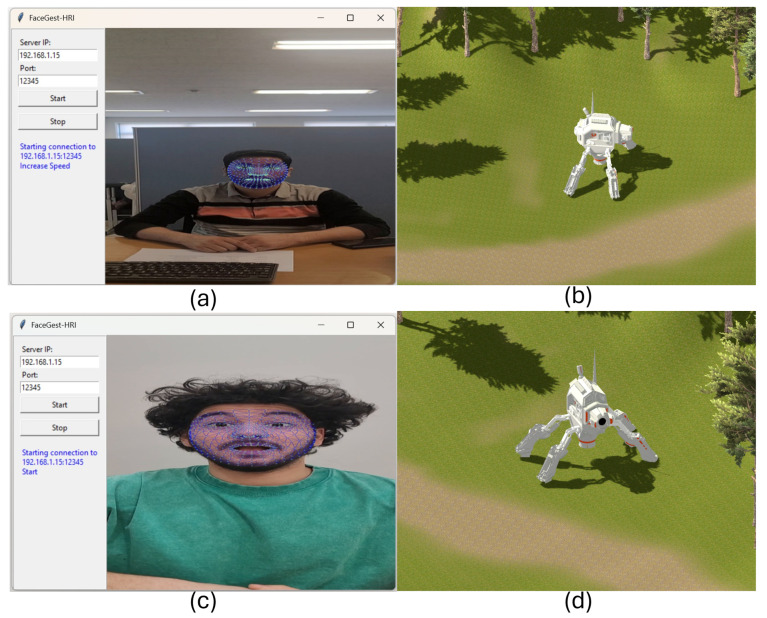
Simulation environment for gesture-based robot control. (**a**) GUI of the FGR (GBA) system showing Subject A performing a ‘raise eyebrows’ gesture. (**b**) Robots receive and execute the corresponding command. (**c**) Subject B is performing a different facial gesture. (**d**) Robot executing the corresponding command, demonstrating real-time interaction.

The proposed method’s simulation environment is implemented in Unity Engine. Figure 3 provides screenshots of this 3D environment and the graphical user interface (GUI) of GBA, which delivers a real-time user experience for ground robot control. The simulator runs on a separate PC and receives commands from the FGR module (GBA) via TCP/IP sockets, as illustrated in Figure 2. The simulator module operates independently, connected over an IP network, and provides constant feedback to the FGR module for each executed command. Robot maneuvers such as moving forward, backward, turning, picking, and dropping objects are performed accurately according to the mapped commands listed in Table 3 and Table 4.

To mimic real-world dynamics, Unity’s physics engine handles inertia, collisions, and force vectors, with each behavior applied according to the specific command. Figure 3 shows example scenarios in the simulator. Figure 3 also illustrates subjects interacting with the remote VR robot using facial gestures. Panel (a) shows the GUI of the FGR (GBA) system, where Subject A is performing a “raise eyebrows” gesture, and the corresponding command is sent to the robot. Panel (b) displays the robot receiving and executing this command. Similarly, Subject B performs a different facial gesture in panel (c), and the robot’s response is shown in panel (d), demonstrating real-time interaction between users and the simulated environment.

### 3.6. Socket Communication

**Figure 4:**
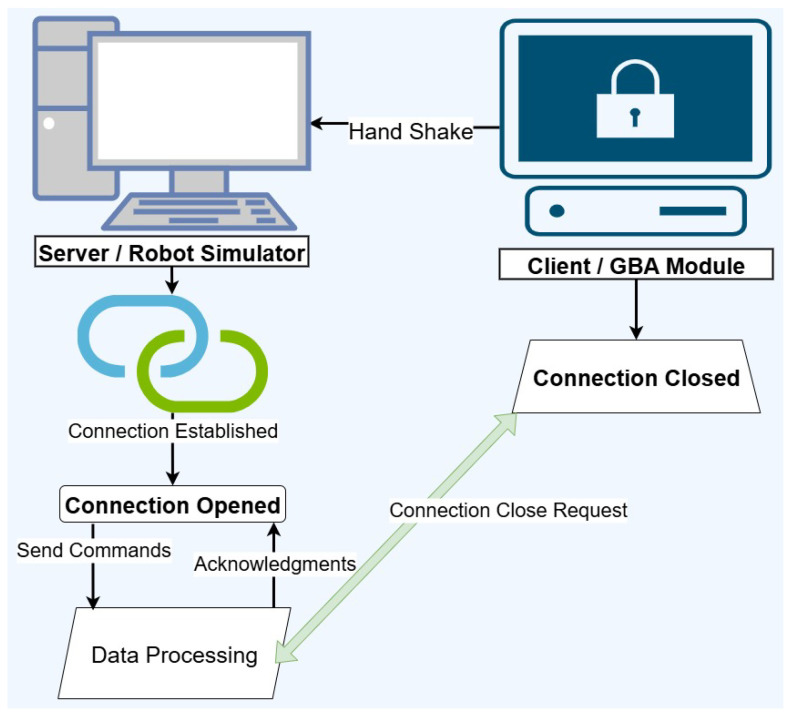
Communication between GBA-Module and the 3D simulator module via sockets.

In our proposed system, the 3D Unity simulator acts as the server, while the FGR (GBA) module functions as the client, as shown in Figure 4. For each command, the client establishes a connection and sends a request to the server. The messaging protocol and key parameters are detailed in Table 2. Upon receiving a command from the FGR module, the server sends an acknowledgment and forwards the command to the robot for execution, ensuring accurate and real-time operation.

### 3.7. Dataset

Most existing benchmarks for facial gesture recognition focus primarily on static images for emotion recognition and typically offer only a small number of gesture categories [4,36,38]. This limitation reduces their suitability for human–computer interaction (HCI) applications, where dynamic, video-based gesture data is essential for capturing temporal variations. To address this gap, we utilize FaceGest [59], a publicly available large-scale dynamic facial gesture dataset specifically designed for interaction-oriented research.

The dataset comprises 13 gesture classes with labeled video samples, collected under diverse lighting conditions and environmental settings to ensure robustness. Each sample captures dynamic sequences rather than single frames, making it suitable for temporal modeling with deep learning architectures. In addition, FaceGest provides pre-extracted deep feature representations to facilitate rapid experimentation.

In our experiments, 10 facial gesture classes were considered, with a total of approximately 15,000 video samples, averaging around 1100 samples per gesture. For each class, 80% of the samples (approximately 880 videos) were used for training, while the remaining 20% (approximately 220 videos) were allocated for testing/validation. Following standard evaluation protocols, we employed a five-fold cross-validation approach, ensuring balanced class distributions across all folds. Table 3 and Table 4 summarize the dataset and command mapping information. Specifically, Table 3 presents the number of training and test samples for each gesture class, while Table 4 lists the gesture class names along with their mapped control commands and class abbreviations.

## 4. Experimental Evaluation

Experiments for the proposed system were conducted to evaluate the performance of vision-based dynamic facial gesture recognition, computational efficiency, and real-time applicability for interactive control tasks. The evaluation was carried out using the publicly available FaceGest [59] dataset, which provides 13 distinct facial gesture classes recorded under diverse conditions. Our focus was on assessing classification accuracy, robustness across different gesture categories, and inference speed in comparison with existing baseline methods.

### 4.1. System Setup and Configuration

The proposed system was evaluated using a cross-platform client–server configuration. The Unity-based 3D simulation environment acted as the server and was deployed on a Windows 11 Home workstation (HP Pavilion Gaming Desktop TG01-1xxx, HP Inc., Palo Alto, CA, USA) equipped with an Intel^®^ Core™ i5-10400F CPU at 2.90 GHz, 32 GB of RAM, and a Realtek Gaming GbE network interface. This server maintained a TCP socket connection to receive gesture commands from the remote client, acknowledged each command, and updated the virtual environment in real time, enabling responsive simulation control. The client consisted of the FGR module (GBA), which was trained and executed on an Ubuntu 22.04.5 LTS workstation with a 3.50 GHz Intel Core i9-10920X CPU, dual NVIDIA GeForce RTX 3090 GPUs, and 32 GB of RAM. The model was trained for 350 epochs with early stopping enabled. Categorical cross-entropy was used as the loss function, and the Adam optimizer was employed for optimal weight updates. The tanh activation function was used in the BiLSTM layers, while softmax activation was applied at the final output layer for gesture classification.

Fifteen subjects participated in testing the proposed system. The FGR (GBA) module was installed on one machine, and the Unity 3D simulator on another, connected through IP addresses as shown in Figure 4. The communication mechanism is illustrated in Figure 3. To evaluate real-world applicability, the simulation was replaced with a physical ground robot and interfaced via an ATmega32U4 microcontroller, enabling the direct execution of commands predicted by the FGR system.

Experimental averaged test results for each class were recorded and are presented in Table 4 and Table 5. Comparisons with existing methods are reported in Table 6 and Table 7. Each subject performed the gestures listed in Table 4. Unlike systems based on wearable gloves or aided sensors, the vision-based gesture recognition allowed subjects to perform gestures naturally without maintaining strict distances or requiring extensive training.

### 4.2. Evaluation of Vision-Based Face Gesture Classification

The proposed GBA pipeline integrates GhostNet for lightweight spatial feature extraction, followed by bidirectional LSTM layers with temporal attention for dynamic gesture sequence modeling. To quantitatively assess performance, we computed classification metrics on the test split of the FaceGest dataset, including precision, recall, F1-score, and overall accuracy (Table 5).

**Figure 5:**
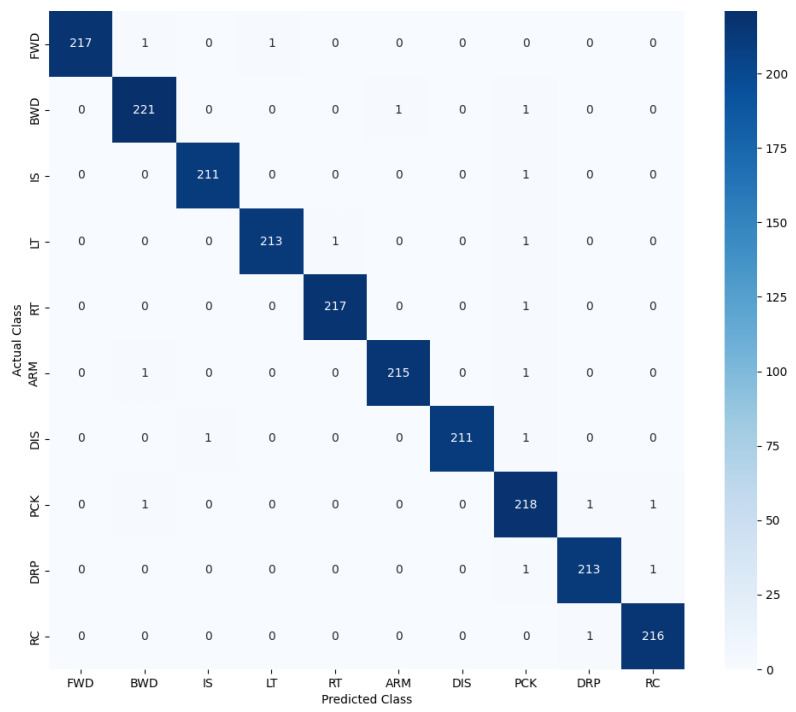
Confusion matrix for vision-based facial gesture recognition trained model based on the test data.

The confusion matrix (Figure 5) demonstrates the ability of our model to distinguish between eye-based, mouth-based, head-based, and combined gestures, with minimal inter-class confusion. Classes with visually subtle differences, such as *blink* versus *double blink*, were also accurately recognized due to the temporal attention mechanism highlighting key frames.

**Figure 6:**
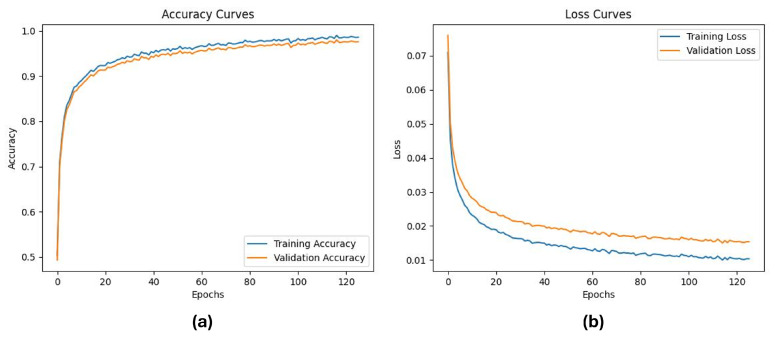
The training and validation curves of the proposed model: (**a**) Accuracy over epochs for training and validation sets; (**b**) Loss value variation over epochs for training and validation sets, showing stable convergence.

To further evaluate the training behavior of the proposed model, we analyzed the variation in accuracy and loss over epochs. As shown in Figure 6, the training and validation accuracy (Figure 6a) steadily increases, while the training and validation loss (Figure 6b) decreases consistently, indicating smooth convergence without overfitting. These curves confirm the stability of the model training process and support the effectiveness of the proposed architecture for dynamic facial gesture recognition.

In comparison with baseline dynamic gesture recognition methods from the literature, our approach consistently achieved higher accuracy and significantly reduced the computational cost, making it suitable for deployment on resource-constrained systems. The evaluation confirmed that the integration of attention modules into GhostNet and LSTM layers improved the model’s robustness in varying lighting conditions and across different subjects.

### 4.3. Evaluation Based on Lap Time

One of the standard performance indicators in gesture-based control systems is the lap time [60,61], which measures the total time taken from the initiation of a gesture to the completion of the corresponding action. The primary objective of the proposed system is to provide a natural, safe, and intuitive interface for controlling robots and UAVs, particularly for users with no prior control experience. Therefore, lap time was selected as a key evaluation metric.

A total of 15 participants took part in the experiments, performing the complete set of facial gestures defined in the FaceGest dataset. The system was evaluated in a virtual simulation environment implemented in Unity3D 6.0. For each participant, the time required to execute the control commands using all gestures was measured and averaged to obtain the lap time (shown in Table 7 and Table 8).

The results demonstrated that the proposed GBA architecture, enhanced with spatial and temporal attention mechanisms, enabled faster and more responsive control compared to existing vision-based gesture recognition systems. The lap times achieved by our system were significantly lower, confirming its suitability for non-expert users. Additionally, the consistently low detection time ensured that commands were processed and executed with minimal delay, resulting in a smoother and more intuitive interaction experience.

Overall, these findings validate that the proposed GBA approach not only improves recognition accuracy but also enhances real-time responsiveness, making it highly applicable to hands-free HCI scenarios such as robotics, UAV navigation, and immersive metaverse applications.

## 5. Discussion

This study presents a vision-based facial gesture recognition framework for the real-time remote control of robotic systems in both virtual environments. The compact gesture set, combined with the system’s low-latency response, enables intuitive operation with minimal cognitive load for users, even those without prior experience. This was achieved by designing a concise set of 10 easily distinguishable gestures, balancing functional coverage with operational simplicity. Such a design ensures reliable control in challenging or time-sensitive scenarios, avoiding the pitfalls of overly complex gesture vocabularies that may overwhelm operators, or overly simplistic ones that limit task execution [68,69].

Compared to existing vision-based interaction systems, the proposed method is characterized by its simplicity, adaptability, and extensibility. While the core command set is intentionally minimal, it can be expanded depending on application-specific needs, which makes the system flexible for deployment across different domains. Furthermore, the integration of socket-based communication enables seamless remote operation within metaverse-style environments, extending the scope of traditional gesture-based control systems. The recognition engine is built on a hybrid GhostNet-BiLSTM backbone, augmented with spatial and temporal attention mechanisms. This architecture combines the efficiency of lightweight convolutional feature extraction with the temporal modeling capacity of recurrent networks, enabling the robust detection of dynamic facial gestures with minimal computational overhead. The model’s responsiveness and temporal pattern recognition capabilities make it readily transferable to other vision-based HCI applications, from robotic teleoperation to immersive VR/AR interaction.

### Limitations

Despite its promising results, the system has certain limitations. The current implementation uses a fixed set of 10 base gestures to ensure ease of use and reduce cognitive burden. While this constraint improves user experience, it may limit control granularity in highly complex scenarios, such as multiple robot operations, where additional derived commands would be necessary. Expanding the gesture set, however, must be approached carefully to maintain usability.

Another limitation lies in the scope of evaluation. The reported results are primarily based on the FaceGest dataset and a real-world pilot study with 15 participants. While the high accuracy values and live testing results are encouraging, they may not fully capture the model’s generalization capability across different datasets or unseen subjects. A cross-dataset evaluation was not conducted in this work due to dataset availability and compatibility constraints, and we explicitly recognize this as a direction for future research.

In addition, while several baselines were included for comparison, not all were re-trained under identical experimental conditions. Some results were adopted directly from prior publications, as shown in Table 6. This distinction may affect the fairness of comparisons, although care was taken to ensure that all baseline results are reported accurately and from reputable sources.

Finally, the current implementation assumes stable computational resources and controlled environmental conditions. Deployment in highly dynamic or resource-constrained settings may require further optimization and robustness checks to ensure consistent performance.

## 6. Conclusions

In this paper, we proposed a lightweight and efficient vision-based facial gesture recognition system for the intuitive remote control of robots in a virtual environment. Leveraging a hybrid GhostNet-BiLSTM architecture with spatial and temporal attention, our method achieves high accuracy and low latency on the FaceGest dataset, demonstrating robust performance across varied lighting and user conditions. The integration with a Unity3D-based simulation environment via socket communication enables seamless, real-time command transmission, offering a novel approach to contactless human–robot interaction.

The compact gesture vocabulary designed in this work strikes a balance between functionality and user cognitive load, making it suitable for both expert and novice users. Experimental evaluations, including gesture classification accuracy and lap time metrics, validate the system’s superiority over existing vision-based control methods in terms of responsiveness and usability.

Future work will focus on extending the gesture set to support more complex control scenarios, including multi-robot coordination. Additionally, we plan to explore adaptive learning techniques to personalize gesture recognition and improve robustness under diverse real-world conditions. Beyond these directions, we also aim to conduct cross-dataset and unseen subject evaluations to better assess the generalization capabilities of the framework. This will help identify potential dataset biases and ensure reliability across different user groups and environments. Another important direction is optimizing the framework for deployment in resource-constrained platforms, such as mobile or embedded systems, where real-time performance and energy efficiency are critical.

Overall, the proposed system paves the way for accessible, immersive, and reliable vision-based interfaces in assistive robotics, teleoperation, and metaverse applications.

## Figures and Tables

**Table 2:** Implementation details and considerations for the socket communication module used in real-time robot control.

Module	Implementation Details	Considerations
Socket Protocol	TCP/IP protocol for robust communication	Ensures command reliability; handles packet loss
	Option to use UDP for low-latency scenarios	Trade-off: potential packet drops
Client–Server Architecture	Server runs the robot simulator; client runs HGR command system	Supports multiple simultaneous client connections
	Heartbeat mechanism to monitor connectivity	Allows reconnection if connection drops
Port Configuration	Fixed port for command transmission	Both client and server must agree on the port
	Dynamic port allocation for multiple robots	Useful in multi-robot setups
Data Format	Commands serialized in JSON byte streams	Ensures structured and human-readable commands
	Optional compression for large data packets	Reduces latency in heavy data exchange
Message Size	Defined buffer size for each command	Prevents overflow and data corruption
	Split large commands into multiple packets	Maintain sequence numbers for proper reassembly
Command Latency	Total round-trip time for command execution	Acknowledge receipt to ensure action completion
	Measure execution delay for optimization	Can log timestamps for performance monitoring
Connection Management	Initialize, maintain, and close socket connections	Automatic retry on failure to maintain continuity
	Keep-alive signals to prevent idle timeout	Monitors connection health and detects interruptions

**Table 3:** Distribution of training and testing samples per gesture class on an average of 1100 samples per gesture.

Class Label	Mapped Command	Training Samples (80%)	Testing Samples (20%)
FWD	Move forward	875	219
BWD	Move backward	890	223
IS	Increase speed	860	215
LT	Turn left	870	218
RT	Turn right	885	221
ARM	Start/activate	880	220
DIS	Stop/deactivate	865	216
PCK	Pick object	895	224
DRP	Drop object	870	218
RC	Rotate camera	880	220

**Table 4:** Mapped commands and accuracy for each class in the proposed facial gesture recognition model.

	Facial Gesture Classes	Mapped Command	Accuracy
Vision-Based FGR	Smile	Move Forward (FWD)	99.0%
	Frown	Move Backward (BWD)	98.9%
	Raise Eyebrows	Increase Speed (IS)	99.2%
	Blink (Left Eye)	Turn Left (LT)	98.8%
	Blink (Right Eye)	Turn Right (RT)	99.3%
	Mouth Open	Start/Activate (ARM)	99.1%
	Puff Cheeks	Stop/Deactivate (DIS)	98.7%
	Lip Bite	Pick Object (PCK)	99.0%
	Wink	Drop Object (DRP)	98.9%
	Head Tilt	Rotate Camera (RC)	99.4%

**Table 5:** Performance metrics of the proposed facial gesture recognition model on the FaceGest dataset.

Mapped Command	Precision (%)	Recall (%)	F1-Score (%)
Move Forward (FWD)	99.2	99.0	99.1
Move Backward (BWD)	98.9	98.8	98.8
Increase Speed (IS)	99.4	99.1	99.2
Turn Left (LT)	98.7	98.9	98.8
Turn Right (RT)	99.3	99.2	99.2
Start/Activate (ARM)	99.1	99.0	99.0
Stop/Deactivate (DIS)	98.8	98.6	98.7
Pick Object (PCK)	99.0	98.9	99.0
Drop Object (DRP)	98.9	99.0	98.9
Rotate Camera (RC)	99.4	99.3	99.4

**Table 6:** Comparison of the proposed GBA method for dynamic facial gesture recognition with baseline algorithms.

Algorithm	Application Domain	# of Classes	Accuracy
Mollahosseini et al. [39]	FER	7	94.5%
Dhal et al. [40]		6	92.8%
Mishra et al. [51]		8	96.5%
Howard et al. [50]		10	97.6%
Jiang et al. [45]		7	98.3%
Wasi [55]		8	96.6%
Li et al. [56]		7	90.5%
Xue [57]		7	90.8%
GBA (proposed)		10	99.13%

**Table 7:** Performance comparison of ViT-based dynamic and facial gesture-based HCI control systems.

Method	Input Modality	Number of Gestures	Lap Time (ms)	Detection Delay (ms)	Processing Speed (fps)
GBA	RGB	10	129.5	18.5	30
Aouayeb [54]		7	244	75	28
Wasi [55]		8	205	83	25
Li et al. [56]		7	190	59	30
Xue [57]		7	259	92	29
Huang [58]		7	234	87	32

**Table 8:** Performance comparison of Hybrid CNN-based dynamic and facial gesture-based HCI control systems.

Method	Input Modality	Number of Gestures	Lap Time (ms)	Detection Delay (ms)	Processing Speed (fps)
GBA	RGB	10	129.5	18.5	30
Zhang et al. [62]		7	157	31	30
Ming et al. [63]		7	163	33	35
Serengil et al. [64]		7	181	41	33
Savchenko [65]		8	174	39	38
Zheng et al. [66]		7	172	35	34
Antoniadis et al. [67]		7	188	39	32

## Data Availability

Project source code is available on request.

## Abbreviations

The following abbreviations are used in this manuscript:

GBA	GhotNet-BiLSTM-Attention
FGR	Facial Gesture Recognition
BiLSTM	Bidirectional Long Short-Term Memory
HRI	Human–Robot Interaction
